# Overcrowding estimation in the emergency department: is the simplest score the best?

**DOI:** 10.1186/cc12196

**Published:** 2013-03-19

**Authors:** V Winkin, V Pinckaers, V D'Orio, A Ghuysen

**Affiliations:** 1 CHU Liège, Belgium

## Introduction

Emergency department (ED) overcrowding is a major international problem with a negative impact on both patient care and providers. Among validated methods of measurement, emergency physicians have to choose between simple and complex scores [1,2]. The aim of the present study was to compare the complex National Emergency Department Overcrowding Scale (NEDOCS) with the simple occupancy rate (OR) determination. We further evaluated the correlation between these scores and a qualitative assessment of crowding.

## Methods

The study was conducted in two academic hospitals and one county hospital in Liège, Chênée and Verviers; each with an ED census of over 40,000 patient visits per year. Samplings occurred over a 2-week period in January 2011, with five sampling times each day.

## Results

ED staffconsidered overcrowding as a major concern in the three EDs. Median OR ranged from 68 to 100, while the NEDOCS ranged from 64.5 to 76.3. We found a significant correlation between the OR and the NEDOCS (Pearson *R *= 0.973, 0.974 and 0.972), as well as between the OR, the NEDOCS and the subjective evaluation (*P *= 0.001).

## Conclusion

Crowding evaluation in the ED requires validated, easy-touse, scores. Our study indicates that the simple OR is as accurate as the complex NEDOCS, allowing continuous crowding assessment.
